# Impact of intercurrent illness on calcium homeostasis in children with hypoparathyroidism: a case series

**DOI:** 10.1530/EC-17-0234

**Published:** 2017-09-06

**Authors:** A Chinoy, M Skae, A Babiker, D Kendall, M Z Mughal, R Padidela

**Affiliations:** 1 Royal Manchester Children’s HospitalManchester, UK; 2 King Abdullah Specialized Children’s HospitalRiyadh, Saudi Arabia; 3 Royal Preston HospitalPreston, UK

**Keywords:** hypoparathyroidism, hypocalcaemia, intercurrent illness, alfacalcidol

## Abstract

**Background:**

Hypoparathyroidism is characterised by hypocalcaemia, and standard management is with an active vitamin D analogue and adequate oral calcium intake (dietary and/or supplements). Little is described in the literature about the impact of intercurrent illnesses on calcium homeostasis in children with hypoparathyroidism.

**Methods:**

We describe three children with hypoparathyroidism in whom intercurrent illnesses led to hypocalcaemia and escalation of treatment with alfacalcidol (1-hydroxycholecalciferol) and calcium supplements.

**Results:**

Three infants managed with standard treatment for hypoparathyroidism (two with homozygous mutations in *GCMB2* gene and one with Sanjad-Sakati syndrome) developed symptomatic hypocalcaemia (two infants developed seizures) following respiratory or gastrointestinal illnesses. Substantial increases in alfacalcidol doses (up to three times their pre-illness doses) and calcium supplementation were required to achieve acceptable serum calcium concentrations. However, following resolution of illness, these children developed an increase in serum calcium and hypercalciuria, necessitating rapid reduction to pre-illness dosages of alfacalcidol and oral calcium supplementation.

**Conclusion:**

Intercurrent illness may precipitate symptomatic hypocalcaemia in children with hypoparathyroidism, necessitating increase in dosages of alfacalcidol and calcium supplements. Close monitoring is required on resolution of the intercurrent illness, with timely reduction of dosages of active analogues of vitamin D and calcium supplements to prevent hypercalcaemia, hypercalciuria and nephrocalcinosis.

## Introduction

Hypoparathyroidism (HPT) is the partial or complete reduction of parathyroid hormone (PTH) secretion from the parathyroid glands. PTH stimulates 1-alpha-hydroxylase enzyme to produce 1,25(OH)_2_-vitamin D, which facilitates active absorption of oral calcium (Ca) from the intestine. PTH also mobilises Ca from the bone (by increasing the number and activity of osteoclasts to encourage bone resorption) to maintain serum Ca concentration. PTH also regulates serum phosphate. HPT is therefore characterised by hypocalcaemia and hyperphosphatemia.

The standard treatment for HPT is with active vitamin D (calcitriol (1,25(OH_2_)D)) or its analogue (alfacalcidol (ACD; 1-hydroxycholecalciferol)) and ensuring adequate oral Ca intake (through diet and supplements if necessary) (1). PTH in addition stimulates renal Ca reabsorption in the distal nephron and therefore in severe HPT increased urinary Ca excretion may occur when serum corrected calcium (cCa) is at the upper end of the reference range. Therefore, historically it has been important to maintain the cCa of these patients below the normal range (between 1.9 and 2.2 mmol/L), and frequently monitor cCa and urinary calcium creatinine ratio (Ur Ca:Cr), along with responsive dose changes to prevent both under-treatment (resulting in symptomatic hypocalcaemia) and over-treatment (resulting in potential for hypercalciuria and nephrocalcinosis) (1). However, we do recognise that the optimal cCa for each patient with HPT often depends on the specific aetiology and genetic mutations.

There is scanty information in the medical literature, which describes the impact that intercurrent illness may have on serum cCa concentration in children with HPT. We describe a case series of three children with genetic causes of HPT in whom intercurrent illnesses led to escalation of treatment with ACD along with Ca supplements. We provide specific advice on management of HPT during intercurrent illnesses.

## Methods

### Ethics statement

This case series did not need approval from an ethical committee, as it simply involved a retrospective review of case notes with no intervention or other investigation.

### Consent statement

Consent has been obtained from each patient or subject after full explanation of the purpose and nature of all procedures used.

## Case series description and results

### Case 1

**Figure 1:**
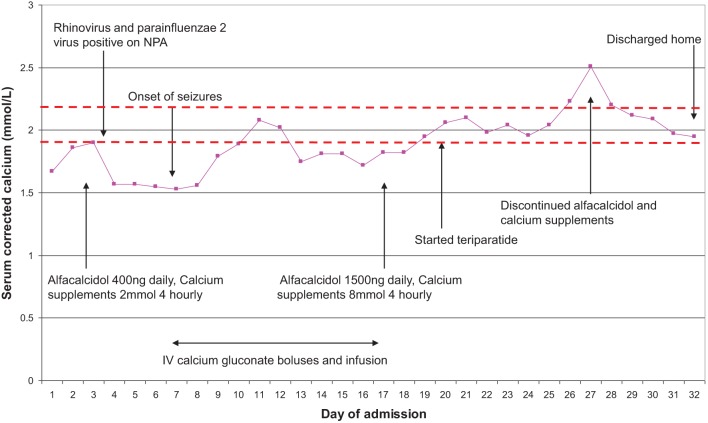
Presentation and progress of case 1 during bronchiolitic illness, demonstrating fluctuations in serum corrected calcium during admission and management needed. NPA – nasopharyngeal aspirate. Dotted lines indicate the range of optimal serum corrected calcium concentration for children with severe hypoparathyroidism (1.9–2.2 mmol/L).

A two-month-old male with HPT due to homozygous *GCMB2* (glial cell missing b 2) mutation had cCa, which was maintained within the normal range for infants with HPT by ACD 400 ng (80 ng/kg) daily and Ca supplements (initial PTH at diagnosis <0.6 pmol/L, normal range 1.6–6.9 pmol/L). He developed bronchiolitis caused by rhinovirus and parainfluenzae virus type 2. On day four of illness, his serum cCa dropped from 1.90 mmol/L at the start of illness to 1.53 mmol/L, which was associated with a hypocalcaemic seizure. This was managed with intravenous calcium gluconate – initially as boluses (0.11 mmol/kg), but thereafter with an infusion (1–2 mmol/kg/day) in view of persistent hypocalcaemia. Over the following 12 days, ACD dose was increased up to 1500 ng (300 ng/kg) daily and Ca supplements increased from 12 mmol (2.5 mmol/kg, 100 mg/kg) daily to 48 mmol (10 mmol/kg, 400 mg/kg) daily. Despite these substantial doses, and tolerance of full enteral milk feeds, control of cCa concentration remained suboptimal (largely 1.6–1.9 mmol/L), with hypercalcuria (Ur Ca:Cr 2.05 mmol/mmol, mean value for children <12 months: 1.50 mmol/mmol (2)) demonstrated with cCa >1.9 mmol/L. Given these challenges, subcutaneous injections of teriparatide – recombinant human PTH(1–34) (rhPTH(1–34) was initiated on an off-licence basis (dose 0.4 μg/kg twice-daily)). This established acceptable cCa for a child with severe HPT (mainly 1.9–2.3 mmol/L) over the following days, allowing discontinuation of ACD and oral Ca supplements within 7 days and discharge within 12 days. Furthermore, hypercalciuria that had been demonstrated whilst on ACD improved (Ur Ca:Cr 0.74 mmol/mmol). Figure 1 displays his cCa during illness, along with interventions instigated. He subsequently has remained on rhPTH(1–34), as it has provided better control of cCa whilst avoiding hypercalciuria.

### Case 2

A two-month-old male with HPT secondary to *GCMB2* mutation was stable on ACD 400 ng (110 ng/kg) daily (initial PTH at diagnosis <0.6 pmol/L). He presented with bronchiolitis due to respiratory syncytial virus and rhinovirus. Despite normal feeding, his cCa dropped to 1.44 mmol/L with resulting hypocalcaemic seizures. He required intravenous Ca infusion and his dose of ACD was increased to 1500 ng (400 ng/kg) daily along with oral Ca supplementation (5 mmol/day, 1.3 mmol/kg/day, 50 mg/kgday). After 11 days of admission, his bronchiolitis resolved, his cCa improved to 2.0–2.2 mmol/L and he was discharged home on ACD 1200 ng and oral Ca supplementation of 5 mmol daily.

**Figure 2:**
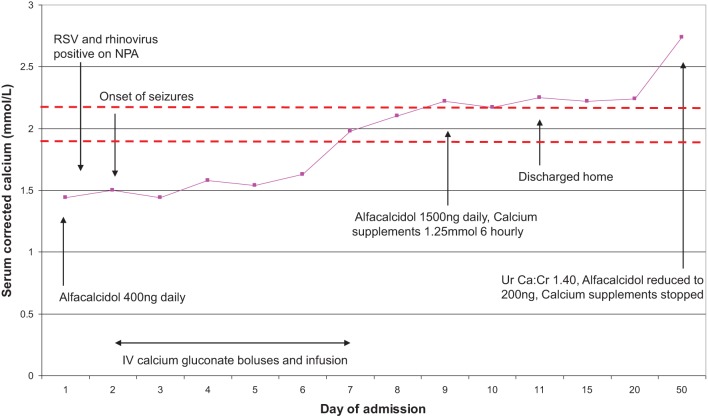
Presentation and progress of case 2 during bronchiolitis illness, demonstrating changes in serum corrected calcium during admission and management needed. NPA – nasopharyngeal aspirate; RSV – respiratory syncitial virus; Ur Ca:Cr – urinary calcium:creatinine ratio. Dotted lines indicate the range of optimal serum corrected calcium concentration for children with severe hypoparathyroidism (1.9–2.2 mmol/L).

During a two-week period of frequent cCa monitoring, his cCa was stable between 2.0 and 2.2 mmol/L. After one month, his investigations revealed hypercalcaemia (cCa 2.74 mmol/L) and hypercalciuria (Ur Ca:Cr ratio 1.40 mmol/mmol). Although this might only be borderline hypercalcaemia in a healthy infant, it is considered significantly elevated in a child with severe HPT due to the risks associated with hypercalciuria. This necessitated discontinuation of oral Ca supplementation and a rapid reduction in ACD doses to 200 ng (55 ng/kg) daily. Figure 2 displays his cCa during and after this illness, along with alterations in management.

### Case 3

A six-month-old male with Sanjad-Sakati syndrome, confirmed by genetic mutation in *TBCE* (tubulin-specific chaperone E) gene, had HPT (PTH <0.6 pmol/L) and was stable on standard doses of ACD, though had already developed signs of nephrocalcinosis. He presented to a tertiary hospital in Saudi Arabia with viral gastroenteritis and subsequent hypocalcaemic seizures. This necessitated increase in doses of ACD up to 3000 ng per day to maintain his cCa within an acceptable range.

Unfortunately, due to distance of tertiary paediatric services, he was lost to follow-up for 18 months on 3000 ng of ACD per day. He represented at 2 years of age with symptomatic hypercalcaemia (cCa >3 mmol/L) and severe bilateral nephrocalcinosis.

Achieving optimal cCa (1.9–2.2 mmol/L) on reduction of ACD proved challenging and he was therefore transferred to our tertiary unit in the UK. Standard doses of ACD were unable to achieve normocalcaemia without worsening hypercalciuria (Ur Ca:Cr 0.7–3.9 mmol/mmol). He was therefore commenced on teriparatide (dose 0.4 µg/kg twice-daily) on which he has been able to maintain his cCa between 2.0 and 2.2 mmol/L with no further progression of nephrocalcinosis (Ur Ca:Cr mainly <0.5 mmol/mmol).

## Discussion

We have reported three cases of HPT where intercurrent illness required escalation in treatment doses of active analogues of vitamin D and oral Ca supplements to maintain adequate cCa levels. There are few reports in the medical literature, which describes loss of control in Ca concentration in patients with HPT during intercurrent illness. Bhadada and coworkers (3) described two adult cases – a 30-year-old female and an 18-year-old male – with HPT who developed an upper respiratory infection and viral gastroenteritis respectively, resulting in hypocalcaemia necessitating increased Ca supplements, with or without increased calcitriol or ACD therapy. To our knowledge, there are no such reports in the paediatric literature.

This case series demonstrates that infants with HPT are susceptible to sudden, severe and symptomatic hypocalcaemia during times of intercurrent illness, despite prior adequate control. This was particularly observed in cases 1 and 2, both of whom suffered hypocalcaemic seizures as a result of their bronchiolitic illness and required marked increase in doses of ACD and oral Ca to achieve optimal cCa concentration. We would therefore recommend vigilance for periods of illness, and to urgently attend health care services if mild symptoms of hypocalcaemia (irritability, lethargy, muscle spasms tremors) or risk factors for deterioration (such as vomiting or poor feeding) are identified. Even without such features, measurement of cCa is strongly advised to ensure no impending hypocalcaemia, which could be corrected with increased intake of oral Ca and ACD or infusion of intravenous Ca. Doing so in a timely manner may prevent more profound symptomatic hypocalcaemia. Parental education regarding the above is crucial to identifying affected children early.

Furthermore, cases 2 and 3 demonstrate that close monitoring of cCa is also needed on resolution of illness in children with HPT. It appears that resistance to standard management resolves on resolution of the illness, necessitating a reduction in active vitamin D and oral Ca doses. Failure to reduce doses will increase the risk of hypercalciuria and nephrocalcinosis as was seen in case 3. Parental education regarding this risk will facilitate frequent monitoring of this complication and weaning regime as appropriate.

All three patients had severe HPT due to genetic causes – two infants had *GCMB2* mutations, which causes severe HPT due to lack of the Gcmb2 transcription factor that is vital for parathyroid gland development (4) and one infant had Sanjad-Sakati syndrome, which also manifests with severe HPT (5). In our experience, this loss of control is less likely to occur in children with partial HPT, for example, due to 22q11.2 deletion syndrome (despite their immunodeficiency as part of this syndrome making them more prone to severe illness) (6), where the resistance to treatment may not be as marked.

Our case series suggests a greater affect on infants with HPT. Although the cause for this can only be hypothesised, a combination of poorer reserves and a greater impact of feed intolerance would seem contributory.

The cause for this transient deterioration in control of children with HPT during intercurrent illness, despite adequate prior control on oral active vitamin D analogues and Ca supplements, remains unknown. Further research would be warranted to elucidate the exact mechanisms. It could be simply that these illnesses influence intake and absorption of dietary Ca and medications, as suggested by Bhadada and coworkers (3). On this point, it is worth mentioning that infants with severe HPT who are kept fasted for prolonged periods may also suffer hypocalcaemia due to inadequate dietary Ca intake.

However, our cases were tolerating feeds during their illness (barring case 1 during early phase of illness). We postulate that this phenomenon may involve cortisol-driven effects on 1,25(OH)_2_-vitamin D. It is recognised that the stress of intercurrent illness is associated with a physiological increase in cortisol secretion. There is evidence to support that cortisol reduces Ca absorption from the gut (7, 8). Furthermore, it has also been demonstrated that this cortisol-induced reduction in Ca absorption is resistant to administration of 1,25(OH)_2_-vitamin D (9). *In vitro* studies in animal models have also suggested that cortisol stimulation may cause PTH release (10). Thus, in an unaffected person and those with partial HPT, cortisol released during intercurrent illness would reduce gut absorption of Ca, but this would be balanced by PTH release, which maintains normocalcaemia. However, in subjects with severe HPT, there is complete lack of PTH release to counter reduced gut Ca absorption, resulting in hypocalcaemia. Perhaps, 'sick day' rules, akin to those well established for children with cortisol deficiency, might need to be applied to ACD and rhPTH doses in children with severe HPT. In the absence of firm evidence to support any specific rules, we advise an individualised case-by-case approach. Our pragmatic practice is to increase ACD dose by approximately 50% and rhPTH dose by approximately 10% at the onset of illness. Thereafter, 6–12 hourly monitoring, either of cCa or ionised Ca, along with Ur Ca:Cr, is essential to adjust doses accordingly to maintain cCa within the optimal range. Furthermore, monitoring of biochemistry on resolution of illness (and rapid reduction of doses to baseline) is equally important, to avoid over-treatment.

Inflammatory cytokines may also have a role in this phenomenon. Interleukin (IL)-1β and IL-6 are both pro-inflammatory cytokines whose levels have been shown to increase within hours of infection in crticially ill patients and are inversely related to serum Ca concentrations (11). Animal models have demonstrated that both of these cytokines upregulate calcium-sensing receptor expression, suppress PTH secretion, are associated with significantly decreased 1,25(OH)_2_-vitamin D levels and appear to drive PTH resistance by downregulating target-organ PTH receptors (11). Therefore, one could postulate that, in children in whom PTH production is already severely affected, further impact on PTH secretion and resistance may explain the significant hypocalcaemia observed in this cohort during intercurrent illness.

rhPTH(1–34) is the active fragment of PTH and is a recognised treatment for HPT in adults, although its original licence is for management of post-menopausal osteoporosis. However, it is unlicensed and very rarely used in children following a report of osteosarcoma risk in growing rat studies (12). Notably, these findings were not substantiated in primate studies (13). All reports to date in human children have not replicated the concerns identified in rat studies and have been shown to be safe (in the short term thus far) and effective in managing HPT, albeit with small case numbers (14, 15). Therefore, rhPTH(1–34) is more recently increasingly being used on an off-license individual basis. In case 1, where the HPT was resistant to management with large doses of ACD and oral Ca, subcutaneous rhPTH(1–34) was shown to be effective in normalising cCa whilst reducing hypercalciuria. We would suggest consideration of rhPTH(1–34) in severe cases of Ca imbalance in children with HPT during intercurrent illness that are refractory to escalation in ACD doses. However, counselling of parents about the possible long-term risks based on animal studies need to be conveyed. Similar to our cases, others have also used rhPTH(1–34) in infants with HPT refractory to standard doses of oral active vitamin D analogues and Ca supplementation (16, 17, 18). We employ a subcutaneous twice-daily dosing regime, which has been shown to be more effective than once-daily regimes (19, 20), although there is evidence emerging to suggest continuous subcutaneous infusion of rhPTH(1–34) via pumps may confer even greater efficacy in terms of steady cCa concentration (21).

In conclusion, we have demonstrated through our case series the potential for sudden, profound and symptomatic hypocalcaemia in infants with HPT during intercurrent illness, requiring vigilance, regular monitoring and timely escalation of ACD and oral Ca dosage. At the same time, resolution of intercurrent illness results in normalisation of cCa, such that close monitoring and dose reduction to baseline is needed to prevent hypercalcaemia and hypercalciuria. rhPTH(1–34) may have a role in these situations. The mechanisms underlying this phenomenon remain unclear, although we speculate whether excess cortisol secretion during intercurrent illness may play a role. Further research is warranted to elucidate the exact mechanisms.

## Declaration of interest

The authors declare that there is no conflict of interest that could be perceived as prejudicing the impartiality of the research reported.

## Funding

This report did not receive any specific grant from any funding agency in the public, commercial or not-for-profit sector.

## Author contribution statement

A Chinoy – Writing and revising manuscript. M Skae, A Babiker, D Kendall, MZ Mughal – Revising manuscript. R Padidela – Revising manuscript and corresponding author.
